# Insights on Cellulose Research in the Last Two Decades in Romania

**DOI:** 10.3390/polym13050689

**Published:** 2021-02-25

**Authors:** Sergiu Coseri

**Affiliations:** “Petru Poni” Institute of Macromolecular Chemistry of Romanian Academy, 41 A, Gr. Ghica Voda Alley, 700487 Iasi, Romania; coseris@icmpp.ro; Tel.: +40-232-217454; Fax: +40-232-211299

**Keywords:** cellulose, Romania, cellulose oxidation, cellulose-based, materials

## Abstract

In the current global context, cellulose fulfills those characteristics that give it clear advantages over synthetic fibers, having a huge potential for substituting fossil-based materials which are polluting and harmful to ecosystems. Research conducted in most laboratories around the world in the field of cellulose is overwhelmingly aimed at industrial needs because features such as renewability and low cost are the most important attributes for economic success. In this global effort, Romanian researchers contribute through achievements that are briefly reviewed in this paper. These refer to the main achievements reported after 2000 in the field of cellulose characterization and cellulose functionalization, as well as the main areas where cellulose-based materials were applied.

## 1. Introduction

The preoccupation with the processing, obtaining and study of cellulose in Romania has very old roots, which take us to the 16th–17th centuries when the main sources for obtaining paper were represented by cotton, linen and hemp. A much more rigorous and precise systematization of what represents the pulp and paper processing industry can be found only at the end of the 19th century, when the technology based on wood resources was adopted for the production of pulp and paper on the Romanian territory, operating at that time in around 15 modern factories. However, the modern era of cellulose processing and research begins only in 1949 at the Polytechnic Institute in Iasi, when the Faculty of Industrial Chemistry in Iasi was reorganized, and the pulp and paper department was created for the first time in Romania within the organic chemistry section. In 1955, the pulp and paper department expanded its profile to become a pulp, paper and man-made fiber technology. Subsequently, through the significant contributions of the founders of this school and their descendants, a valuable school was created, recognized internally and internationally [1]. A significant role in the creation, organization, development and worldwide affirmation of the Romanian school of pulp and paper was played by Cristofor Simionescu, an illustrious professor and researcher. Several handbooks regarding the chemistry and structure of some wood species or seaweed in Romania appeared in those years, being translated into several foreign languages [2,3,4,5].

**Figure 1:**
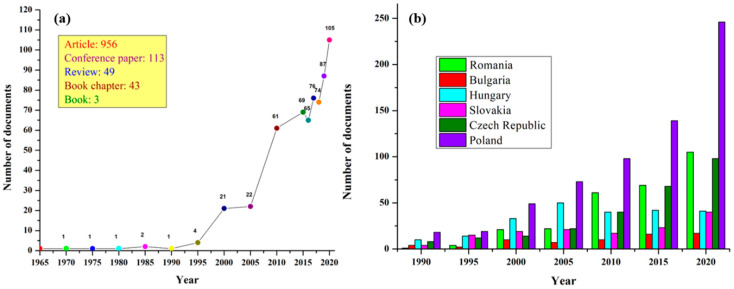
The number of documents versus year published by Romanian researchers (**a**) and researchers from different Central and Eastern European countries (**b**), according to the Scopus database using the keyword “cellulose”.

The special interest shown by Romanian researchers for research in the field of cellulose can be exemplified by publishing: since 1966, a journal dedicated entirely to this research topic, “Cellulose Chemistry and Technology”, ISI indexed from 1992 until 2019. In an attempt to have as complete an image as possible related to the study of cellulose conducted by Romanian researchers, we used the Scopus database, conducting a search using two fields: one related to the word “cellulose” in a search field type (article title, abstract, keywords), and the second search field being related to the affiliation country, using “Romania”. As Figure 1a shows, following this search, a total of 1164 documents were retrieved, including scientific and conference papers, reviews, book chapters and books. It seems surprising that the scientific production in the field of cellulose research is quite modest for a long time: from 1965 to 1990, only one or two documents per year were found to be indexed by the Scopus database, Elsevier, Amsterdam, The Netherlands.

Intrigued by these observations, we tried to see what the results of a similar search were, replacing “Romania” with other countries in the region, such as Bulgaria, Hungary, Poland, Slovakia or the Czech Republic. The author noticed somewhat the same trend with a relatively modest number of publications in the period mentioned above (1965–1990). The extremely reduced number of scientific publications in the field of cellulose in all Central and Eastern European countries can be explained by the low interest in fundamental research during the communist years, society being focused rather on specific industries and research being based more on contracts with industry, which were not completed with scientific publications.

Somewhat paradoxically, with the fall of communism and the beginning of the drastic deindustrialization that affected the pulp and paper industry after 1990 (see Figure 1b), a boost can be seen in the number of published documents related to cellulose. A steady increase can be observed in the number of scientific papers published each year in the field of cellulose, with a maximum of 105 published papers reached in 2020 in Romania. Poland holds the supremacy in terms of the number of published documents since 1990; Romania being in the second position in tandem with the Czech Republic. This growing interest in the basic research directed toward cellulose can be explained due to the alignment of the mentioned countries toward modern research topics, as in Western Europe and the USA, based on translating researchers’ interest into searching for alternative energy resources which are renewable, abundant and cheaper, but also by awareness of the finite nature of oil, combined with the big issues of pollution and contamination which traditional resources cause. Thus, by increasing the financial resources allocated by the European Union to projects aimed at this field, the interest of researchers has also greatly increased, leading to a high scientific production.

**Figure 2:**
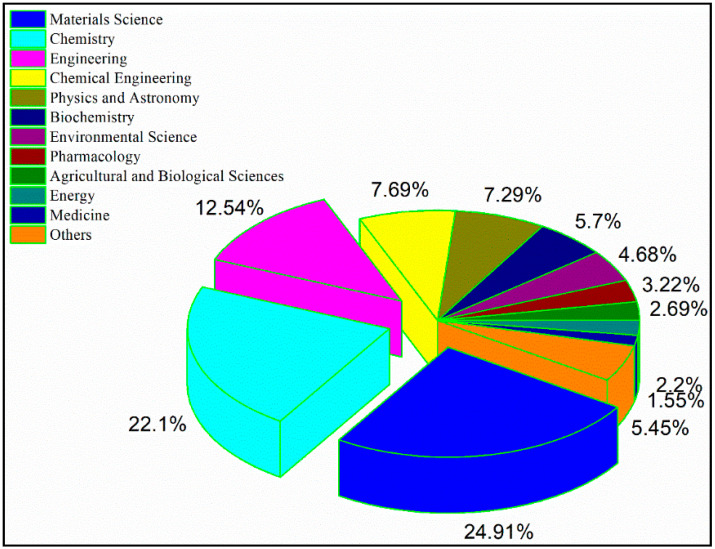
Subject area classification of the scientific production made by Romanian researchers in the period 1965–2020, organized by fields according to the Scopus database using the keyword “cellulose”.

Figure 2 presents the classification of scientific papers published by Romanian authors in the period 1965–2020 according to the field of research. “Materials science” and “chemistry” dominate the interest of researchers, which is understandable given the many challenges posed by cellulose in terms of elucidating its chemical structure and properties and physical properties that are not yet fully elucidated, but also due to the multiple uses of cellulose in materials science, especially in the last ten or fifteen years.

Other subject areas covered by the papers authored by Romanian researchers include engineering and chemical engineering, physics, biochemistry, environmental science, pharmacology, agricultural and biological sciences, energy and medicine.

**Figure 3:**
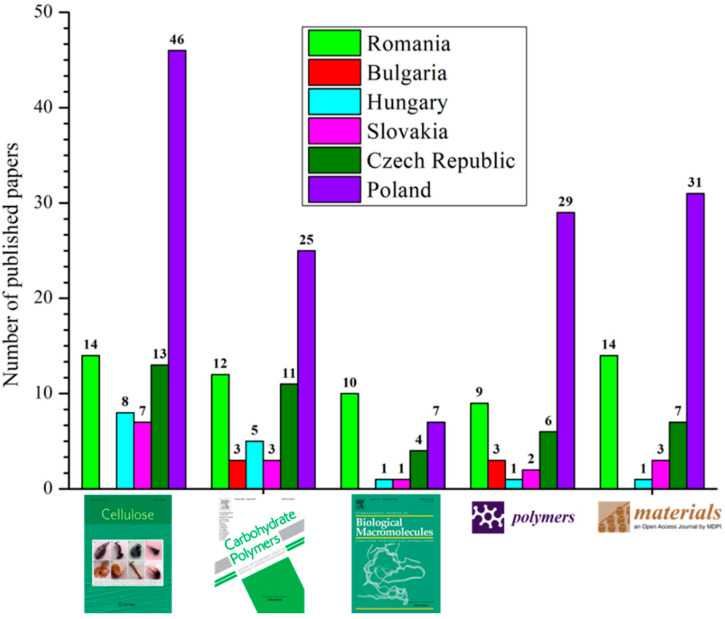
Comparative representation of the total number of published papers in selected journals: Cellulose, Carbohydrate Polymers, International Journal of Biological Macromolecules, Polymers and Materials, by researchers affiliated in Romania and other several countries in Central and Eastern Europe in the period 2015–2020 according to the Scopus database using the keyword “cellulose”.

In order to quantify the impact of the scientific papers published by the authors with affiliation in Romania, the author conducted a study using as a basis the years of publications appearing in the period 2015–2020, indexed in the Scopus database. Thus, the first five journals (to be classified in Q1 and Q2 according to Clarivate Analytics Web of Science^TM^ Journal Citation Report, Philadelphia, PA, US, representative for the “cellulose” research field) in which most articles were published were retained. The following journal titles were identified: Cellulose, Carbohydrate Polymers, International Journal of Biological Macromolecules, Polymers and Materials. Then, these journals were also checked for researchers with affiliation in other Central and Eastern European countries, the results being depicted in Figure 3. Interestingly, the percentage of articles published in the five selected journals as compared to the total number of documents published in the mentioned period is almost the same for the authors affiliated in Hungary, Czech Republic and Slovakia, around 8%, lower for authors affiliated in Bulgaria (about 6%), while Poland and Romania are in the top position with about 12.5% publications.

The purpose of this mini review was to bring to the reader some of the most important achievements of researchers in Romania published after 2000, but also to consider the global research context of the targeted fields. It will thus be easy to observe the main areas in cellulose research approached by researchers affiliated in Romania, such as studies of wood composition, physical properties and functionalization reactions, with a strong emphasis on selective oxidation reactions, but also the concern to introduce cellulose base materials in new and innovative emergent applications.

## 2. Chemical Composition Study of Some Tree Species, Structure and Characterization of Amorphous Cellulose

An exhaustive study on the chemical composition of some tree species from the Northern part of Romania for the period 1964–2000 was conducted. The study was based on the investigation of wooden tree rings in a dominant tree species, *Quercus robur* L., in terms of its chemical composition and structure of natural polymeric components—cellulose and lignin. This study used chemical methods to separate the main components of wood, but also analysis techniques such as Fourier transform infrared (FTIR) spectroscopy and thermogravimetry [6]. The authors of this study made an interesting correlation between environmental and climatic factors and the chemical structure of wood in order to anticipate the possible uses of wood. By analyzing the main regions of FTIR and the characteristics of wood components in the frequency ranges of 3400–2900 cm^−1^ and 1730–1640 cm^−1^, substantial differences were found in the composition of wood due to possible interactions between chemical groups (OH, C=O). At the same time, differences in the crystallinity index of cellulose in oak were found. Differences were also found using thermogravimetric analysis: namely, the thermal decomposition of cellulose is different depending on the height of the shaft and the radial growth. Another investigation that led to an interesting and worrying conclusion at the same time was that which was carried out on sawdust from oak wood. Chemical analyses have shown a high content of mineral elements (ash) compared to a previous study conducted in 1964. These changes may indicate an intense process of drying and degradation of oak wood—a phenomenon currently observed in other ecosystems in Europe in which oak is found [6]. A pioneering work in the field of structure and characterization of amorphous cellulose was carried out by Ciolacu and Popa [7]. In their study, the authors propose a new solvent system for the regeneration of three types of cellulose: microcrystalline cellulose, dissolving pulp and cotton, composed of sulphur dioxide, diethylamine and dimethylsulfoxide. After solubilization and regeneration in ethanol, each cellulose sample was analyzed using X-ray diffraction (XRD); FTIR spectroscopy and differential scanning calorimetry (DSC) were used to determine the crystallinity degree. It has been found that this solvent system does not affect the supramolecular structure of cellulose since the measured values were almost unchanged. Moreover, the viscometric measurements of the cellulose solutions showed that apparently the degree of polymerization of the cellulose used was preserved by dissolution, recording only minor depolymerization processes [7].

## 3. Bacterial Cellulose, Production and Characterization

Cellulose, the most abundant polysaccharide, can be produced by plants and microbial cells, and even by some marine species, i.e., tunicates. Cotton and woods are the main sources for cellulose production. Plant-derived cellulose is naturally stable and non-toxic. It has been speculated that cotton can provide cellulose, which usually appears pure. Cellulose from wood coexists with other components, such as lignin and hemicelluloses. In order to separate pure cellulose, these must be extracted by using a chemical pulp and then through laborious purification procedures. Unlike plant cellulose, some acetic acid bacteria can provide one of the purest forms of cellulose, also called “microbial cellulose”, or “bacterial cellulose” (BC) [8]. BC is an intriguing material which has been extensively researched in last decade due to its spectacular properties such as high purity, excellent mechanical properties and good thermal stability. Although it is practically the same substance, BC has superior physical properties to other types of cellulose obtained from plants. An illustration of the main differences between the properties of the two types of cellulose can be found in Table 1.

**Figure 4:**
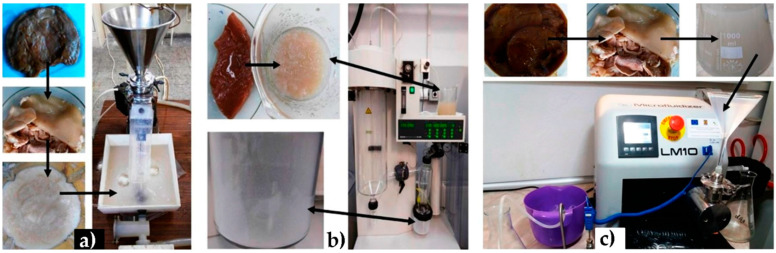
Main steps in purification of nanocellulose (NC): (**a**) purification, grinding with a blender and deep grinding with a colloidal mill; (**b**) purification and atomization using a spray-dryer; (**c**) purification and microfluidization at pressures over 1300 bar. [20].

Due to its remarkable properties, it is not at all surprising that BC has aroused the interest of researchers in Romania. One research group from National Institute for Research and Development in Chemistry and Petrochemistry Bucharest (ICECHIM), in cooperation with researchers from two companies, reported the preparation of cellulose nanofibrils using Kombucha membranes (KM)—a side product resulting from the beverage industry by tea-broth fermentation using coactive cultures of bacteria and yeast [20]. In their study, nano and microfibrils of cellulose were prepared using extensive purification processes of KM previously prepared from tea infusion and sweetener containing contain 50% fructose, 44% glucose and 6% oligosaccharides, inoculated with fermented Kombucha tea (10%) then added to a coactive culture made of bacteria (*Komagataeibacter* and *Gluconobacter*), yeasts (*Zygosaccharomyces, Brettanomyces/Dekkera* and *Pichia*) and lactobacteria. In this way, granules of BC are produced within 30 days, which need further laborious steps of purification realized with sodium hydroxide aqueous solutions in order to remove the fermentation residues, including saccharides, proteins and amino acids. Three processes were then employed to further disintegrate the membranes: microfluidization, atomization and colloidal milling (see Figure 4) [20].

In their study, the authors focused more on the purification steps and use several techniques to assess the success of the purification, concluding that more studies are required for optimizing the purification process approaching greener methods in order to deeper understand the BC biosynthesis [20]. Another study conducted at ICECHIM focused on the isolation of cellulose nanocrystals using a side product from food industry; namely, plum seed shells [21]. To obtain nanocrystalline cellulose, two approaches were used, both using two stages: the difference being in the first stage, when either alkaline hydrolysis or hot-water extraction was used, the second common stage being acid hydrolysis. Following these treatments, the authors revealed that they obtained cellulose nanocrystals with dimensions between 100 and 800 nm in length and about 14 nm in width. Another parameter that has undergone radical changes following the treatments performed refers to crystallinity. If the initial sample had a crystallinity of 38%, the obtained nanocrystalline cellulose had a current crystallinity between 51 and 54% due to the elimination of hemicellulose and lignin [21]. Despite the use of by-products from the food industry, some observations could be made, especially regarding the rather large size of the nanocrystals which are closer to the micro zone (0.8 μm) and the relatively low crystallinity, requiring future studies to improve these two deficiencies. However, the current tendencies for the BC preparation which tries to avoid its production from a chemically defined media as it is an expensive approach thus limits the potential implementation at a larger scale. Moreover, because the BC process takes place anaerobically, the microbial cells are prone to contamination by other microorganisms [22]. Recent innovative alternatives for the BC synthesis consider the utilization of a cell-free system derived from a single cell line [22]. Even if the production of bio-cellulose registered slight variations during the synthesis stages, the overall efficiency of the process was clearly superior to the classic BC production processes which create the premises for the industrialization of the process. An interesting study on this matter was recently published aiming to investigate the synthesis and self-assembly of cellulose nanofibrils in an in vitro environment through intermediate phase analysis, which was compared to the microbial cell system [23].

## 4. Chemical Functionalization of Cellulose

Cellulose (C_6_H_10_O_5_)_n_, the most abundant organic polymer, is the major constituent of plant cell walls, being composed of 1,4-linked β-D-glucose residues (glucose units bounded by ether covalent bound at C_1_ position of one glucose to C_4_ of another glucose). In plants, cellulose is formed from sugar. It serves as building material in the formation of primary cell walls, the other constituents of the primary cell walls being hemicellulose and pectin. It is well known that due to its particular structure, with a complex intra and inter molecular hydrogen bonds network, cellulose is not soluble in water or organic solvents, preventing its use in classical homogeneous reactions of organic chemistry or in applications in which a homogeneous dispersibility with other components can be ensured. In an attempt to overcome these impediments, researchers often resort to cellulose derivatives: compounds that are obtained by functionalization–derivatization reactions performed on cellulose, which bring new properties most often aimed at obtaining soluble derivatives in organic solvents, or ideally in water, as a requirement of green chemistry. The most common processes for achieving these goals are the esterification, etherification and oxidation reactions [24].

### 4.1. Phosphorous Containing Cellulose Derivatives

**Figure 5:**
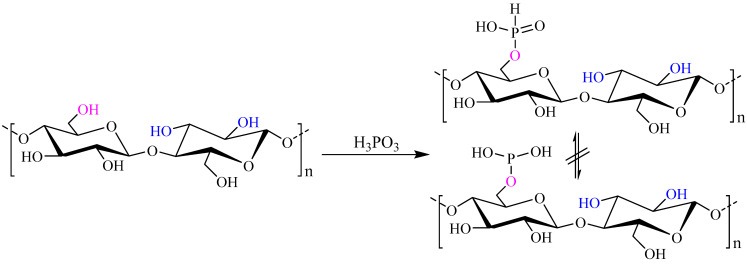
Reaction scheme of cellulose phosphorylation using phosphorous acid. Adapted with permission from Ref. [25]. Copyright 2006, Elsevier.

Phosphorus can be covalently attached to the cellulose network by a reaction occurring at hydroxyl groups, providing phosphate (Cell–O–P(O)(OH)_2_), phosphite (Cell–O–P(OH)_2_) or phosphonic acid (Cell–P(O)(OH)_2_) groups. The most commonly used reagents to obtain cellulosic derivatives are compounds of pentavalent phosphorus, i.e., H_3_PO_4_, P_2_O_5_, organic phosphates and POCl_3_. Suflet et al. [25] reported the preparation of phosphorylated cellulose by reacting microcrystalline cellulose dissolved in molten urea with phosphorous acid, see Figure 5. 

It was found that the primary OH groups in cellulose were the most reactive, allowing the formation of monobasic cellulose phosphate, Cell–O–P(H)(O)OH, rather than cellulose phosphite (Cell–O–P(OH)_2_), see Figure 5 [25]. It should be noted also that the degree of substitution (DS) of the formed products, as determined by elemental analysis and titration methods (potentiometric and conductometric), was around 1, which determined the water solubility of the product, but at the same time maintained a good thermal stability up to 200 °C.

### 4.2. Oxidation Reaction for the Cellulose Derivatives Preparation

**Figure 6:**
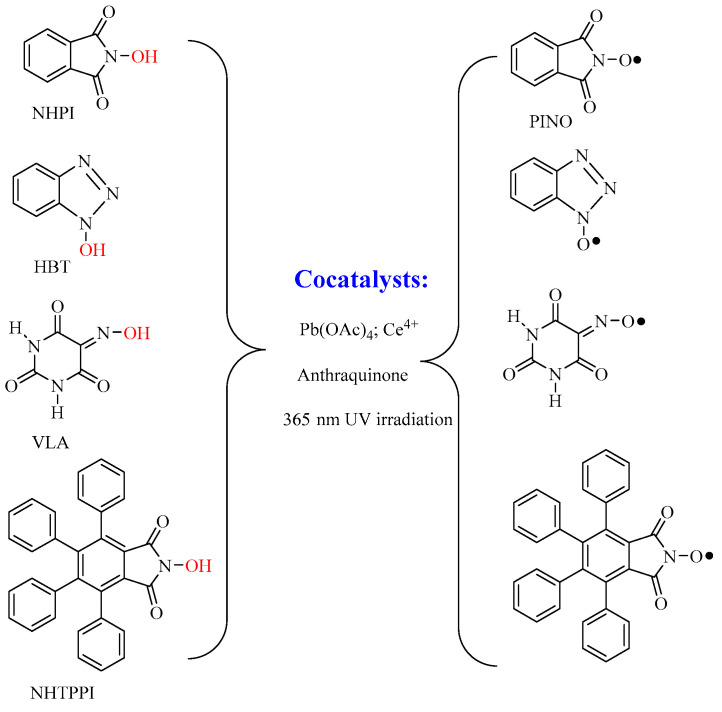
The non-persistent nitroxyl radicals used as mediators for the C_6_ oxidation of cellulose in the presence of sodium hypochlorite and sodium bromide at pH = 10 and room temperature.

The oxidation reaction of cellulose has been extensively studied and well documented within the “Petru Poni” Institute of Macromolecular Chemistry in Iasi in the department led by Sergiu Coseri. Since 2009, they have reported for the first time a new protocol for the selective oxidation of primary alcohol groups in the anhydroglycoside unit of cellulose, using nonpersistent nitroxyl radicals as reaction mediators generated in the reaction medium, in combination with diluted solutions of sodium hypochlorite and sodium bromide at pH = 10 and room temperature [26]. The nitroxyl radical used as a mediator, phthalimide-N-oxy (PINO), was obtained by homolytic cleavage of the >N-O-H bond from N-hydroxyphthalimide (NHPI), see Figure 6. The cleavage of the O-H bond from PINO was possible due to including in the reaction a so called cocatalyst, which promotes the catalytic cycle, and usually contains salts of transition metal salts, anthraquinone, but also UV irradiation at 365 nm.

**Figure 7:**
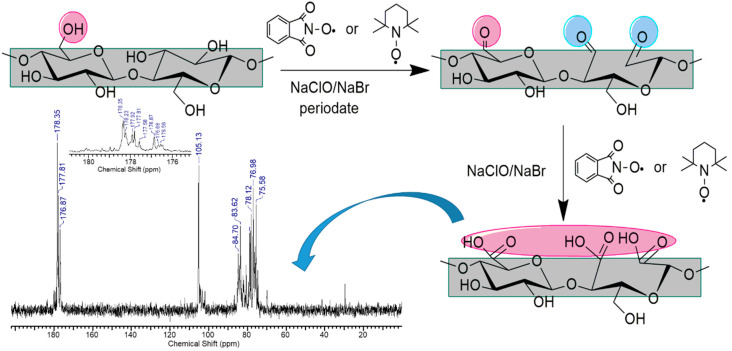
Illustration of the simultaneous oxidation of the three OH groups from the anhydroglucose unit in cellulose performed due to the presence of TEMPO (NHPI) radical and sodium periodate. Reprinted with the permission from Ref. [35]. Copyright 2015, RSC.

This new protocol has been rapidly extended by considering other compounds, such as 1-hydroxybenzotriazole (HBT), violuric acid, (VLA) and N-hydroxy-3,4,5,6-tetraphenylphthalimide (NHTPPI), which are able to generate in situ the corresponding nitroxyl radicals, see Figure 6 [27]. By using these compounds, it has been shown that the degree of oxidation of cellulose can be “adjusted” by using a certain type of nitroxyl radical precursor, a different type of cocatalyst or by varying the reaction time. Later, the oxidation protocols were constantly improved by reporting a bromide-free oxidizing system for the selective oxidation of cellulose, the harmful action of the bromide reagents being well known [28], and also by the utilization of molecular oxygen instead of sodium hypochlorite [29]. The justification for the introduction of new nitroxyl radicals as mediators for the cellulose oxidation reaction as an alternative to the oxidation protocol that uses the stable radical 2,2,6,6-tetramethyl piperidine N-oxy (TEMPO) was also made by the same research team in several original reports and review papers [30,31,32,33,34], with emphasis on the disadvantages of using TEMPO radical, i.e., high cost price, the oxidation reaction proceeds with a high degree of depolymerization of the products and the separation and recycling of the catalyst are difficult to achieve. Even by applying these proposed oxidation protocols, a major shortcoming persists; namely, the resulting oxidation products have lower oxidation degrees, insufficient for the solubilization of the resulting products in water. This is a major impediment to applications in which cellulose could be used, making it difficult to process and compatibilize with various additives. In an attempt to find a solution to these issues, Coseri et al. [35] ingeniously proposed an oxidation method that combines two protocols into one by simultaneously oxidizing all three OH groups in the anhydroglycoside unit with TEMPO or PINO radical (the primary OH groups) and sodium periodate (the two secondary OH groups), see Figure 7.

Applying this one-shot protocol, it was possible to obtain an amount of negatively charged groups as high as 3110 mM/kg cellulose as determined by potentiometric titration within 4 h at pH = 10.5. Another very recent study reported the preparation of water-soluble oxidized cellulose using the TEMPO-mediated oxidation protocol and extended reaction times (up to 24 h). In this way, the authors managed to isolate three fractions of oxidized cellulose: fractions that differ in their degree of oxidation and their solubility in water [36].

## 5. Preparation and Characterization of Hybrid Materials Based on Cellulose

### 5.1. Composite Materials Containing Various Cellulose Types

One of the most common uses of cellulosic materials, including nanocellulose, nanocrystalline cellulose, microfibrillary cellulose, etc., is in the field of composite materials. Polymeric composites containing cellulosic components are intriguing materials possessing unique properties which makes them different from the traditional materials currently available, such as superior strength, easy processability and geometrical complexity at a nanolevel scale, low density and abrasivity, biocompatibility and biodegradability. One study performed at the Centre of Organic Chemistry of the Romanian Academy of Bucharest presented the preparation of new polymeric composites made from polypropylene and two types of cellulose microfibrils [37]. The as prepared composites show enhanced mechanical properties, and the DSC measurements reveal significant changes in melting and crystallization temperatures of the polypropylene matrix when cellulose microfillers and compatibilizer were added. Frone et al. reported the preparation and characterization of polymer composites from polylactic acid (PLA) and cellulose fibers obtained by (i) acid hydrolysis of microcrystalline cellulose (HMCC) or (ii) applying mechanic treatments of regenerated wood fibers, which allow the disintegration (MF) [38]. One of the biggest issues of composite preparation using “ex situ” synthesis methods concerns the inhomogeneity of the components. In order to increase the compatibility between the matrix (PLA) and cellulose fibers (total mass added was 2.5%), the authors performed an additional surface treatment using 3-aminopropyltriethoxysilane (APS). In this way, it was demonstrated by using scanning electron microscopy that a better adhesion between the matrix and cellulose was realized. Moreover, the prepared composites exhibited a superior storage modulus of PLA in the glassy state, which notably increased after silanization, even at higher temperatures [38]. A further study of the same research group on PLA–cellulose nanocomposites focused on the influence of the thermal properties of PLA, as well as its morphology using both untreated and silane-treated cellulose nanofibers as fillers [39]. The thermal properties were analyzed in depth using a repetitive DSC analysis session (heat–cool–heat–cool), and the morphology of both PLA and nanocomposites was investigated using a new AFM technique, Peak Force QNM, which emphasized the nanolevel features by elastic modulus mapping. Recently, the structure of PLA and its nanocomposites with cellulosic nanofibers was studied by combing the annealing effect, as well as the differences between untreated and surface-treated cellulose. The thermal and mechanical properties were also compared for PLA and the resulting nanocomposites [40]. It has been found that the incorporation of the cellulose nanofibers in the PLA matrix led to a Tg diminution independent of the heating rate applied in the DSC analyses, but the Tg value was almost the same as that of PLA in the case of the cellulose nanofibers treated with silane, conforming the enhanced compatibility of the two components. To further confirm the efficiency of the silane treatment of the cellulose nanofibers, XPS and EDX techniques were employed as direct methods, as well as mechanical analyses as an indirect method. Young’s modulus and tensile strength values were higher in the case of the PLA nanocomposites with silane-treated cellulose nanofibers than in the case of the composites with untreated cellulosic nanofibers, while the crystallinity decreased in the case of the PLA-silane treated cellulose nanocomposites, as revealed by DSC and XRD analyses, presumably because of better interfacial adhesion between the components which lowers the PLA chain mobility. Composites of polypropylene with various biomass fillers, such as *Eucalyptus globulus*, Norway spruce (*Picea abies*) thermomechanical pulp, energy grass, *Brassica rapa*, pine cones and grape seeds, were studied using thermogravimetry and in situ ascertaining of the major decomposition compounds, combined through Fourier transform infrared (FTIR) and mass spectrometry (MS) techniques [41]. Analyzing the FTIR/MS spectra, the authors revealed that the principal gaseous products resulting from the degradation of polypropylene/biomass composites are H_2_O, carbon dioxide, carbon monoxide, methanol, formaldehyde, methane, acetic acid, aldehydes, phenols, furans, guaiacols, catechol originating from the biomass decomposition and a complex mixture composed of different hydrocarbons resulting from the polypropylene matrix decomposition [41]. Hybrid nanocomposite materials prepared from ZnO and BC were prepared using the matrix-assisted pulsed laser evaporation (MAPLE) technique [42]. ZnO nanoparticles with an average size of 20–30 nm were synthesized from zinc acetate and ammonia, recovered by centrifugation and then deposited onto BC films in a vacuum chamber by means of an external laser source. Differences of the nanoparticle distribution into the BC matrix are attributed to the solvent used for the ZnO dispersion, i.e., water or chloroform, and also the physical state of the matrix (wet or dry). ZnO nanoparticles deposited on BC were tested against *B. subtilis* and *E. coli*, demonstrating a very good antibacterial activity, but also a good biocompatibility with human dermal fibroblastic cells. The required quantities to achieve these results was 1.68 mg ZnO nanoparticles/mm^2^, which correspond to a 300 nm ZnO nanoparticle coating. Nevertheless, the authors noted that even a very thin layer of ZnO has the capacity to completely inhibit bacterial growth (Gram positive and Gram negative) without a detectable effect on eukaryotic cells. BC represented also an ideal template for calcium phosphate deposition, using its successive immersion in solutions of Ca(NO_3_)_2_·4H_2_O and (NH_4_)2HPO_4_ under intensive ultrasonication. Taking this opportunity, Busuioc et al. [43] reported the preparation of various calcium phosphate structures after calcination of the cellulosic template at a temperature of 600–1200 °C. The authors obtained different types of architectures, ranging from spherical structures composed of nanometric grains or sheets tightly tightened in the radial direction, to structures with high porosity consisting of strongly bound three-dimensional grains. Apart from the calcination temperature, the heating rate and the calcination period have a strong influence on the porosity and grain sizes. Interestingly, the calcium phosphate architectures were loaded with magnetic components, showing that the resulting materials have a reduced specific magnetic momentum, between 0.7 and 4.5 emu/g. These quite small values are explainable due to the high porosity of the samples, which lead to a lower interaction between the grains. Nevertheless, this magnetic load is still useful in such applications where a large and uniform surface area of the magnetic entities in the host environment is demanded without the risk of clumping due to magnetization. The same research group reported that 3D mineral scaffolds based on calcium phosphates can be successfully fabricated by combining the wet-chemistry deposition of the CaCl_2_ solutions on BC membranes, followed by a thermal treatment at a temperature of 700 or 1000 °C [44]. In this case, by analyzing the phase composition the authors reveal that before calcination the structures belong to monoclinic brushite (CaPO_3_(OH)·2H_2_O) and presumably different amorphous phosphates, which are converted after calcination at 700 °C (or 1000 °C) to orthorhombic buchwaldite (NaCaPO_4_) and hexagonal hydroxyapatite (Ca_5_(PO_4_)_3_(OH)) [44]. It is worth noting that these biomaterials, made by using renewable cellulose as a template under relatively simple conditions, can be viable and robust alternatives for development of fillers in areas such as new types of cement or bone scaffolds, offering huge possibilities for exploitation in practice.

### 5.2. Hydrogels Containing Cellulosic Components

**Figure 8:**
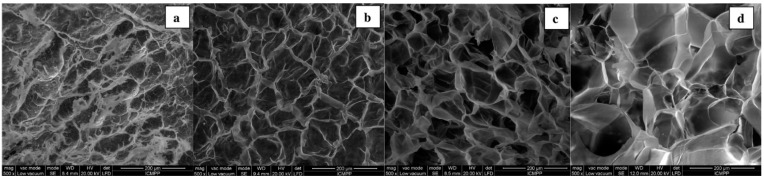
SEM images of cellulose cryogels: (**a**) physically cross-linked, R0 and chemically cross-linked (**b**) using 1 nmolECH/nmolAGU, (**c**) using 2 nmolECH/nmolAGU and (**d**) using 4 nmolECH/nmolAGU. Adapted with permission from Ref. [46]. Copyright 2016, Elsevier.

Research in the field of hydrogels, defined as three-dimensional polymer networks capable of absorbing impressive amounts of biological fluids or water, is a topic that enjoys today great interest from scientists. Research in the field of hydrogels is also due to the high degree of applicability of these materials, a considerable number of uses being already reported in the food industry, health, care and cosmetics, substitutes for various organs (skin), water depollution (dyes and heavy metals removal), sensors and biosensors and many others [45]. The peculiar behavior of cellulose II hydrogels prepared in the presence of epichlorohydrin (ECH) as crosslinking agent was investigated by Ciolacu et al. [46] They prepared both physical and chemical hydrogels (using different amounts of ECH) by dissolving cellulose (5% wt) in aqueous sodium hydroxide solutions (8% wt), followed by coagulation in water and freeze-drying. According to the recorded SEM images in Figure 8, the physical hydrogels show a highly heterogeneous morphology, (Figure 8a), but for the chemically linked hydrogels, the morphology tended to become foam-like, with the disappearance of the denser regions and a bigger pore size (Figure 8b,c, respectively).

**Figure 9:**
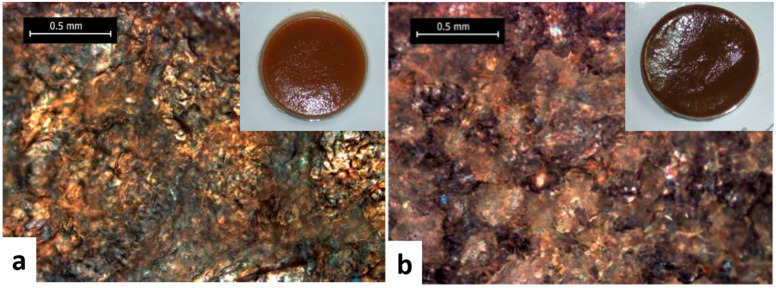
Typical optical photographs of hydrogels made from different mass ratios of cellulose–lignin: 1:1 (**a**) and 1:3 (**b**). The photo images of the hydrogels are presented inset. Adapted with permission from Ref. [47]. Copyright 2013, Cellulose Chemistry and Technology.

The hydrogel with the highest amount of ECH incorporated, 4 nmolECH/nmolAGU, (Figure 8), has again an irregular morphology, presenting denser regions, but pore sizes reaching 200 μm. The differences between the physical and chemical hydrogels synthesized are also related to their different degrees of crystallinity: physical hydrogels have a higher degree of crystallinity than chemical hydrogels, whose crystallinity decreases with increasing ECH content. The authors observed that physical hydrogels contract during the coagulation process, becoming opaque, while chemical hydrogels swell and become transparent. The amount of crosslinking agent (ECH) added to the hydrogel has a strong impact on the degree of swelling and density of the hydrogels. These two parameters increase with an increasing amount of ECH, so the degree of swelling of the hydrogels varies between 700 and 2500% [46]. The explanation given by the authors of these phenomena is related to the hypothesis that ECH acts as a spacer between cellulose chains, which has the effect of disrupting the process of self-assembly of cellulose (much more “visible” in the case of physical hydrogels), leading to a much more homogeneous chemical network and having a high degree of swelling and transparency in the water, but also a considerably low density in the dry state. A combination of cellulose and different ratios of lignin was employed to fabric hydrogels with great potential to be applied in medicine, since it is well known that lignin has great antiviral activity due to a direct interaction with a certain virus, being more efficient against the HIV virus than flavonoids or tannins [47,48]. Lignin inclusion in the cellulose hydrogels leads to a brownish color, see Figure 9, but also to a macro-porous structure with the pores’ diameter ranging from 169–431 μm.

The water vapor sorption values are higher for the hydrogels with increased amounts of lignin, which determine at the same time a reduction of the hydrogel crystallinity index from about 62% (in neat cellulose hydrogel) to about 45% in the cellulose–lignin (mass ratio, 1:3) hydrogel as determined by X-ray diffraction analysis. As a particular class of hydrogels, cryogels refer to such hydrogels constructed at negative temperatures, providing in this way special features required in bioengineering and biotechnological applications [49]. Cryogels containing polyvinyl alcohol (PVA) and microcrystalline cellulose (10–50% *w*/*w*), obtained by freezing/thawing technique, were studied by Paduraru et al. [50], PVA is a synthetic polymer, very convenient to be applied in the preparation of hydrogels because it is water-soluble, non-toxic, biodegradable, biocompatible and possesses remarkable film-forming capabilities. Each repeating unit of PVA contain one hydroxyl group which induces strongly hydrophilic properties of the polymer, and a hydrogen bonding character conferring a high ability to form crosslinked hydrogels. In their study, the authors conclude that the addition of cellulose in the PVA matrix leads to important morphological changes with increasing cellulose content, aspects captured by SEM microscopy, while the cryogel crystallinity can be controlled by adjusting the biopolymer content. Another important role played by the cellulose content in the cryogel is the improvement of the mechanical properties, which is highlighted by the rheology measurement, and also the increased swelling capacity with the increase in the amount of added cellulose [50]. PVA has also been used to prepare self-healing hydrogels in combination with various amounts of tricarboxy cellulose [51,52]. An interesting behavior in the case of using tricarboxy cellulose was observed: according to rheological measurements, the addition of 10% cellulose derivative into the PVA matrix resulted in maximum values of the moduli G′ and G″ concomitantly with a minimum value of the loss tangent due to the strong physical interactions that occurred between the two components. However, the increase in the cellulose tricarboxy content of the hydrogel (up to 25%) has no positive impact; on the contrary, the viscoelastic parameters are drastically affected, for example, the G ′ value of this hydrogel is only 450 Pa, compared to the hydrogel made of neat PVA (500 Pa).

**Figure 10:**
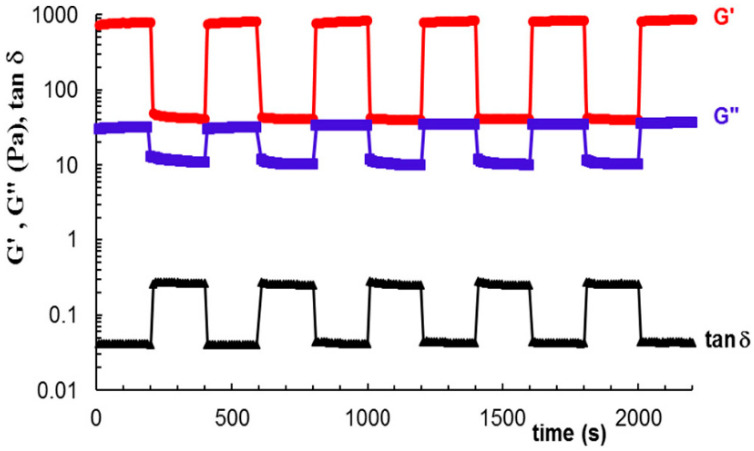
Self-healing behavior illustration for hydrogels prepared from PVA and tricarboxy cellulose (10% *w*/*w*) demonstrated by applying successive deformations of 1% and 200% (ω = 1 rad s^−1^, 25 °C). Reprinted with the permission from Ref. [51]. Copyright 2019, Elsevier.

Oscillatory shear measurements were performed to detect the self-healing ability of the hydrogels. G′, G″ values and tan δ behaviour of the hydrogel sample incorporating 10% tricarboxy cellulose in PVA subjected to consecutive low and high strain deformations are shown in Figure 10, highlighting the self-healing capacity of this kind of hydrogels. The destruction of the sample after exposure at a high shear strain followed by its fast recovery can be explained by taking into account the existence of a highly dynamic network in a permanent equilibrium due to physical interactions. By very quickly restoring the structure of the hydrogel as soon as the external force is removed, the hydrogels return to their original structure and functionality, proving undoubtedly their self-healing character [51].

## 6. Applications of the Cellulose-Based Materials

The intense and continuous preoccupation of researchers for obtaining new cellulose derivatives which possess superior properties, but also in the field of preparation and characterization of modern hybrid materials that incorporate cellulose in different forms, has undoubtedly led researchers to find immediate applications of these materials. It is not surprising that cellulose-based materials, both in their pure state and in different mixtures with various constituents, have found their usefulness in a very wide range of applications, some of which are summarized below.

### 6.1. Dyes and Metallic Ions Removal from Aqueous Solutions

The continuous development of modern society based on consumption and technological processes that use more and more dyes in the textile, leather and paper industries fully contribute to the discharge of large quantities of residues resulting from the manufacturing process in industrial waters. Water pollution is a global issue which tends to reach a crucial crisis, especially in less developed countries that do not have sufficient material resources to procure sophisticated facilities and equipment capable of ensuring the cleaning up of polluted water. Researchers are trying to help communities of people by finding simpler, cheaper, less expensive solutions and quickly implementing in practice the results of research coming from laboratories. In this context, cellulose, being the most abundant natural polymer, has been tested for the removal of various dyes from wastewater. For example, Suteu et al. [53] proposed viscose and its TEMPO-oxidized derivative as an efficient adsorbent of two types of dyes: anionic Brilliant Red HE-3B (BRed) and cationic Methylene Blue (MB). The authors showed that the dye retention depends on certain parameters, such s contact time, dye concentration, pH, adsorbent dose, dye and adsorbent type: the best results being found in the case of MB adsorption on oxidized viscose fibers (454.54 mg/g) [40]. Another study performed at “Gheorghe Asachi” Technical University, Iasi, used another cellulose type, i.e., never-dried softwood bleached sulfite pulp, both pristine and selectively oxidized to introduce carboxylic groups using a TEMPO-mediated protocol. The cellulosic materials were tested as a potential adsorbent for BRed [54]. Following this study, the optimum conditions to attain the highest sorption capacity (between 36–240 mg/L) were found: pH = 1.3, 2 g/L sorbent dose, 15–20 °C and contact time of phases shorter than 400 min. Heavy metals are also a permanent source of water pollution: the presence of these metals being particularly harmful to both humans and living things that live in water. Chromium is one of the most dangerous metals found in water. It can be found in many forms, such as hexavalent chromium, but also in the form of chromate and dichromate. Unfortunately, all these forms are extremely toxic to all life forms, with proven carcinogenic and mutagenic action. BC membranes equipped with magnetic nanoparticles were prepared and tested for the Cr(VI) removal according to the Box–Behnken Design (BBD) [55]. The conclusions of the authors are that chromium removal efficiency is influenced drastically by the pH of the initial solution and also by the interaction between initial Cr(VI) concentration and solution pH. One of the important issue is the magnetization stability of the cellulose membranes, which could suffer from iron leaching, especially at very low or very high pH values. The reported optimum pH for Cr(VI) adsorption onto cellulose–Fe_3_O_4_ membranes was 4.

### 6.2. Applications of TEMPO-Oxidized Cellulose

#### 6.2.1. UV Shielding Materials

**Figure 11:**
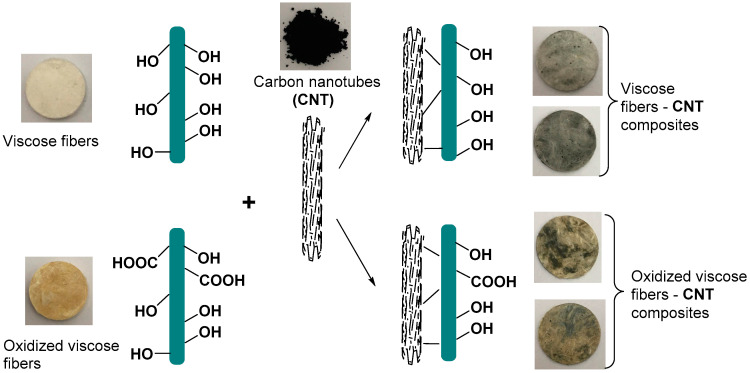
The visual appearance of the composites obtained from viscose fibers and oxidized viscose fibers and carbon nanotubes, using ultrasonication. Adapted with permission from Ref. [56]. Copyright 2019, Wiley.

A recent study performed by Culica et al. [56] shows that viscose fibers in their natural form or oxidized at C6 to introduce carboxyl groups are able to embed carbon nanotubes using ultrasonication process, see Figure 11. The resulting composites possess electromagnetic shielding properties, especially in the industrial frequency range (50–55 Hz).

It is worth to note that the shielding values (dB) for all prepared composites were significantly higher (60–110 dB) than those of other similar materials at industrial frequencies. Moreover, the ease of the process and the natural resources (cellulose fibers) utilized could be beneficial to envisage new and efficient applications. A different type of UV shielding material has been proposed in the form of transparent films made of cellulose acetate and various amount of cerium oxide nanoparticles [57]. In order to obtain a better stabilization and attachment to the polymer matrix, the authors performed a functionalization of cerium nanoparticles with 3-aminopropyl (diethoxy) methylsilane, before incorporating them into cellulose acetate matrix, thus introducing amino groups capable of better stabilization and attachment to OH and cellulose acetate groups by hydrogen bonding. Different degrees of protection against UV radiation can be achieved by simply varying the concentration of nanoparticles embedded in cellulose acetate films.

#### 6.2.2. Contrasting Agent for Noninvasive Cellular Imaging and Magnetic Resonance Imaging (MRI)

**Figure 12:**
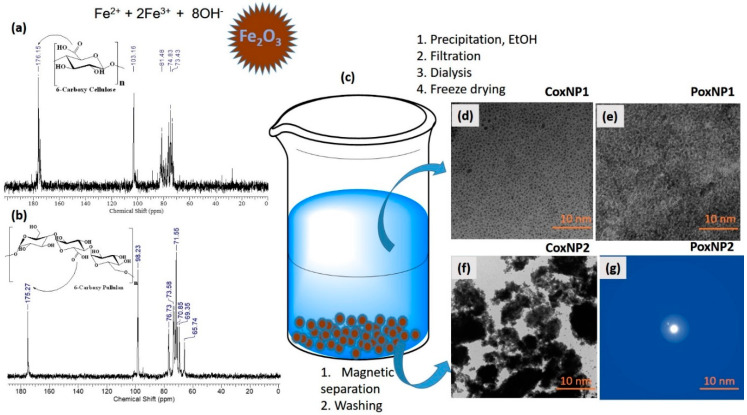
The in situ use of the carboxylated polysaccharides (cellulose or pullulan) to prepare Fe_3_O_4_ nanoparticles surrounded by polysaccharide matrix: the ^13^C-NMR spectra of 6-carboxy cellulose, emphasizing the COOH peak at 176.15 ppm, (**a**); the ^13^C-NMR spectra of 6-carboxy pullulan, emphasizing the COOH peak at 175.27 ppm, (**b**); schematic representation of the Fe_3_O_4_ nanoparticles preparation, (**c**); TEM images of the nanoparticles obtained upon precipitation of the carboxylated polysaccharides (**d**,**e**); and magnetic separation (**f**,**g**). Reprinted with permission from Ref. [58]. Copyright 2017, Wiley.

A useful and nonconventional application of the carboxylated cellulose has been reported recently [58]. In their report, the authors expand the knowledge in the field of oxidized cellulose towards its use as a contrasting agent in MRI. Such use becomes possible by combining an assortment of cellulose with a high degree of oxidation, which gives the material a good water solubility, followed by the inclusion in the cellulose structure of magnetic nanoparticles, freshly prepared in situ (Figure 12).

The prepared materials exhibited promising capabilities to be applied as antibacterial agents against Gram-positive bacteria and also as contrasting substances for noninvasive cellular imaging and MRI. Another type of cellulose substrate (oxidized viscose fibers) has been used to immobilize Fe_3_O_4_ nanoparticles for the preparation of magnetic materials with potential applications in the fabrication of special secured papers or biomedicine [59].

#### 6.2.3. Proton Conductive Composites Based on Carboxyl Cellulose

In recent years, research has heavily focused on finding alternative energy sources that no longer depend on traditional fuels. Researchers’ efforts have been directed towards fuel cells, a technology viewed with great optimism to cover future energy needs. Fuel cells, due to their high efficiency and low emissions, are reliable technological solutions worth considering in the global approach to achieve a low-carbon environment. In this context, the design, practical realization and quick implementation of fuel cells is today a fundamental and strategic field of research in many laboratories around the world. In a typical fuel cell, chemical energy is transformed into electricity, following elementary and reversible reactions which are based on the exchange of protons. One of the key elements of fuel cells on which the successful operation of these elements is based is the electrolyte, i.e., the part that ensures the separation between the anode and the cathode. A study conducted at the Institute of Macromolecular Chemistry “Petru Poni” in Iasi proposed the use of oxidized cellulose mixed with a series of three heterocyclic compounds, such as 1-hydroxybenzotriazole (HBT), imidazole (IM) and 7-azaindole (AI) to be applied for the construction of new proton separation membranes in fuel cells [60]. The authors found that the transport of protons along hydrogen-containing heterocyclic molecules can be compared in terms of efficiency and conductivity with that of well documented sulfonated membranes, but in the proposed membranes, a great advantage is the higher melting point of heterocyclic molecules, which ensures a more stable and robust operation for a greater temperature range. These hybrid materials have been tested under anhydrous conditions up to functional values close to 200 °C, and it is estimated that structures such as imidazole, HBT and azaindole, incorporated in the oxidized cellulose matrix, are suitable for the realization of proton conductive membranes in fuel cells.

## 7. Conclusions and Future Outlooks

Undoubtedly, the research in the field of cellulose will continue to develop rapidly. There are still aspects related to the complex structure of cellulose; its physical properties have not yet been fully elucidated. That is why the author considers that interdisciplinarity and close cooperation between chemists, physicists and biologists has an important role. Sustained efforts are needed to find new methods of functionalizing cellulose using more environmentally friendly reaction protocols and processes that allow obtaining cellulose derivatives which are easier to process and soluble in organic solvents or even water. Another direction of research that can bring significant improvements refers to the use of cellulose for the manufacture of new materials, with applicability in cutting-edge fields such as electronics, medicine and pharmacy. From this point of view, hybrid, composite materials play an important role. All scientific discoveries in laboratories, in order to be applied on an industrial scale, require the preservation of the valuable characteristics of cellulose, such as its biodegradability and non-toxicity. Unfortunately, a number of applications still face many challenges to be implemented industrially. For example, ionic liquids successfully used on a laboratory scale to prepare nanocellulose on a laboratory scale are still prohibitive for industrial processes due to their very high cost. Hybrid materials containing cellulose can be extended for industrial use, but processing specifications play a significant role in this regard. In composites, achieving a homogeneous and uniform dispersion of nanocellulose in a matrix is another challenge.

## Figures and Tables

**Table 1:** A comparison of some properties for plant cellulose (PC) and bacterial cellulose (BC), according to Wang et al. [9].

Property	PC	BC	Reference
Tensile strength (MPa)	25–200	20–300	[10]
Young’s modulus (MPa)	25–200	Sheet: 20,000 Single fibre:130,000	[11]
Water holding capacity (%)	25–35	>95	[12]
Size of fibers (nm)	micrometer scale	20–100	[13]
Crystallinity (%)	40–85	74–96	[14]
Relative hydrophilicity (%)	20–30	40–50	[15]
Purity (%)	<80	>99	[16]
Degree of polymerization	300–10,000	14,000–16,000	[17]
Porosity (%)	<75	>85	[18]
Total surface area (m^2^/g)	<10	>150	[19]

## Data Availability

Not applicable.
